# Applying regenerative medicine techniques in facial plastic and reconstructive surgery: the bar has been set high

**DOI:** 10.1038/s41536-017-0025-0

**Published:** 2017-06-19

**Authors:** Matthew Q. Miller, Stephen S. Park, J. Jared Christophel

**Affiliations:** UVA Health System, Box 800713, Charlottesville, VA 22908 USA

A recent review article examined the current and potential future uses of the latest regenerative medicine techniques in facial plastic and reconstructive surgery (FPRS).^1^ In a follow-up interview, the authors were asked why FPRS as a specialty has been slower to adopt cutting-edge regenerative medicine techniques. Two explanations were proposed: first, in general, the outcomes in the FPRS specialty are already excellent. With facial reconstruction, faster healing, higher rates of graft take, and lower rates of wound infection typically occur than in other parts of the body. These outcomes historically have been explained by the “privileged” blood supply of the face. The second reason why newer regenerative medicine techniques have not become widely-adopted is that the face is a “high-rent” district of the body; there is no margin for error. Even small imperfections on the face are obvious in day-to-day interactions. Given the excellent healing of the face and the demand for excellent outcomes, is there room for the potential benefits of regenerative medicine in FPRS?

One advantage that regenerative medicine techniques could bring to FPRS is the development of engineered cartilage. The ability to tissue engineer cartilage would significantly augment the armamentarium of surgeons for septorhinoplasty because autologous cartilage is of limited supply, especially in revision procedures. Procedures such as major auricular reconstruction require harvesting costal cartilage, which adds morbidity and risks to the reconstructive procedure. Work has already begun on potentially improving distraction osteogenesis—a common method of reconstructing congenital craniofacial anomalies—using bone marrow mesenchymal stem cells (BMMSCs) in animal models.^2^ Cell-assisted lipotransfer, a technique in which centrifugation is used to concentrate adipose-derived mesenchymal stem cells (ADSCs) in fat grafting, has been widely adopted by facial plastic surgeons as it preserves volume and symmetry after facial augmentation procedures.^3^ More research is required to test the efficacy and safety of these methods before pursuing clinical trials in human patients.

The use of stem cells for craniofacial regeneration is another underdeveloped area for regenerative medicine in FPRS. Developmental syndromes such as Treacher Collins, Goldenhar, and DiGeorge exhibit craniofacial abnormalities that originate from dysfunctional neural crest cell survival and/or migration. Unfortunately, cranial neural crest cells (CNCCs) are extremely limited in supply in adults which limits their use for tissue regeneration. Utilizing what is known about cell fate decision pathways could prevent or treat these conditions with directed stem cell therapy. Historically, the majority of craniofacial tissue regeneration clinical and animal trials have used mesenchymal stem cells, specifically BMMSCs and ADSCs. While BMMSCs do partially originate from CNCCs, more work is required to clarify how well they can recapitulate the original “craniofacial stem cell” when used for craniofacial tissue regeneration. For example, in animal models, there are differences in the bone quality produced by BMMSCs and CNCCs.^4^ In a recent study, Zhao et al. showed that these Gli1+ mesenchymal cells were indispensable to craniofacial bone development and repair in mice.^5^ Another recent study found that periodontal ligament stem cells—a known source of mesenchymal stem cells in adults—co-express neural crest cell markers in appropriate culture conditions, demonstrating their neural crest lineage and suggesting they might still be able to function as CNCCs in the right extracellular conditions.^6^ There has been mixed results in the limited animal studies using BMMSCs and ADSCs and the few case reports of using stem cell therapy in humans. The inability of these adult stem cell populations to recapitulate the properties of CNCCs—the original “craniofacial stem cell”—could explain the inconsistent results. More work needs to be done in the lab to compare stem cell populations and the ability to heal and regenerate craniofacial tissues.

Regenerative medicine techniques are presently not able to compete with the current dependable methods of FPRS. One way for regenerative medicine to gain traction in the specialty could be the refinement of regenerative medicine techniques to make them more clinically practical than current standards of care. For example, free tissue transfer produces great results with regards to reconstruction of craniofacial defects from ablative oncologic surgery and complex trauma. However, these procedures typically last 6–8 h or longer and require prolonged admission with typically some time spent in an intensive care unit setting. Regenerative medicine techniques could be an attractive alternative to free tissue transfer if they reduced the operative times and/or postoperative recovery time. The INTEGRA^TM^ bilayer wound matrix dressing is a relative success story where a collagen-glycosaminoglycan matrix can be used to reconstruct large full-thickness scalp wounds that traditionally required free tissue transfer.^7^ The INTEGRA^TM^ dressing has allowed surgeons to perform reconstruction on patients who are unable to tolerate traditional free tissue transfer reconstruction due to comorbidities.

In the past decade, FPRS surgeons have largely attempted a “shotgun-type approach” in testing regenerative medicine techniques. Specifically, different studies have used different combinations of scaffolds, growth factors, and/or stem cells to try and regenerate craniofacial tissues. This approach has led to case reports and small case series but very few large case series and clinical trials to determine if results are equivocal, or potentially superior to current standards of care. The challenge of introducing regenerative medicine techniques into clinical practice is not unique to FPRS. In a recent editorial, Badylak and Rosenthal suggest that perhaps our ability to 3-D print biosynthetic scaffolds, differentiate stem cells in vitro, use growth factors to enhance natural healing processes, and utilize other regenerative medicine technologies has gotten ahead of our understanding of the natural developmental processes that occur.^8^ Improving our understanding of developmental biology and the biology of tissue repair will serve as a road map for developing tissue regeneration strategies for FPRS.
